# Editorial: Updates and New Concepts in Regulation of Proinflammatory Gene Expression by Steroid Hormones

**DOI:** 10.3389/fendo.2018.00191

**Published:** 2018-04-27

**Authors:** Isaias Glezer, Cristoforo Scavone, Maria Christina W. Avellar

**Affiliations:** ^1^Department of Biochemistry, Escola Paulista de Medicina, Universidade Federal de São Paulo, São Paulo, Brazil; ^2^Department of Pharmacology, Instituto de Ciências Biomédicas, Universidade de São Paulo, São Paulo, Brazil; ^3^Department of Pharmacology, Escola Paulista de Medicina, Universidade Federal de São Paulo, São Paulo, Brazil

**Keywords:** inflammation, gene expression, chromatin remodeling, nuclear receptor, glucocorticoid, mineralocorticoids, cardiosteroids, selective glucocorticoid receptor agonists

**Editorial on the Research Topic**

**Updates and New Concepts in Regulation of Proinflammatory Gene Expression by Steroid Hormones**

Inflammatory reactions and their associated molecules contribute to the progression and persistence of several human pathologies. Modern medicine developed successful pharmacological strategies to inhibit or decrease inflammation. Glucocorticoids (GCs) acting on their cognate nuclear receptors (GRs) represent the leading strategy for almost completely shut down inflammation, but the side effects are of major concern regarding long-term use of steroidal anti-inflammatory drugs. Significant advances in the field of molecular biology helped to clarify the “genomic” effects mediated by GRs on DNA transcription in immune cells, establishing a new paradigm of inflammatory response regulation. Recently, our knowledge reached a much higher level of complexity, which could not be completely translated yet to the medicine field. In order to uncover how endogenous GCs and other steroidal molecules, including sex hormones, naturally shapes the immune response, researchers have explored the ways some nuclear receptors modulate gene expression programs involved in proinflammatory signaling. This research topic invited articles that focused on highlighting the specific effects of steroid hormones on inflammatory signaling of a particular biological system or describing recent advances on the current knowledge in terms of signal mediation or mechanism.

The GC corticosterone (CORT) can be further metabolized to aldosterone (Aldo) in the adrenal cortex, a closely related molecule that signals through mineralocorticoid receptor (MR). It is well established that Aldo/MR plays an important role in blood pressure regulation through sodium retention at the kidney level. Growing evidences point toward a different scenario for MR effects on vascular cells and macrophages, including the increased levels of proinflammatory molecules and leukocyte inflammation. This effect can contribute to the pathogenesis of atherosclerosis and hypertension (Moss and Jaffe). Blunted MR signaling seems to be beneficial during brain injury by avoiding exaggerated immune cells responses. MR binds to CORT with higher affinity than Aldo and increases the complexity of neuroinflammation regulation in the brain of stressed animals; a condition that elevates substantially the levels of GCs. GR behaves paradoxically during prolonged and disturbed GC levels and seems to cooperate with a proinflammatory effect of GCs in the brain (Duque Ede and Munhoz). Neuroinflammation is recognized as an important component associated with the progression of various neurodegenerative diseases. Dietary interventions, such as excessive calorie intake and intermittent fasting, modulate GCs and sex hormones and could impact the overall gene expression profile of proinflammatory molecules in the brain during injury or neurodegenerative diseases. While the former statement remains to be verified in its totality, it is well established that estrogen and testosterone modulates gene expression in stimulated immune cells (Vasconcelos et al.). Although it was not possible to include all variety of steroidal hormones in this research topic, the contribution relating cardiotonic steroids (CTS) and the modulation of inflammation is noteworthy. Unlike most of the actions of the aforementioned hormones, CTS bind to and modulate Na^+^, K^+^-ATPase (NKA pump) and do not modulated transcription through nuclear receptors (Orellana et al.). Only very recently an unusual interplay between CD36, NKA, and *Toll*-like receptor 4 revealed the link between CTS and nuclear factor NF-κB activation, a key transcription factor of proinflammatory genes (1). Overall, we may expect that steroid hormones research on inflammation continues to challenge us with new mechanisms and old contradictions.

In terms of mechanisms, three review articles focused on GR-mediated control of proinflammatory gene expression. Simplifying GR actions, the nuclear receptor can induce the transcription of genes that encode anti-inflammatory proteins, or interfere with transactivation activity on proinflammatory genes, or signal through “non-genomic” pathways. Glucocorticoid-induced leucine zipper (GILZ) is an example of the first case. GILZ interferes with proinflammatory signaling in leukocytes at transcription factor level, which is well documented in lymphocytes (Ronchetti et al.). GR interference on transcription activity can operate through different modalities, including tethering and changes in the chromatin state. In fact, GR tethering is the basis of the generation of a new pharmacological interference strategy in inflammation that employs selective glucocorticoid receptor agonists. Diverse mechanisms have been proposed to explain GR effects on proinflammatory transcription, which can include time-dependent dynamics (Xavier et al.). Finally, GR can operate non-canonically, and interferes with inflammation *via* “non-genomic” mechanisms through second messengers or *via* a mitochondrial GR (Scheschowitsch et al.). The many faces of GR actions on proinflammatory transcription are still under debate and recent findings reveals unpredicted mechanisms, such as rapid and prolonged chromatin decompaction promoted by the nuclear receptor (2). Future studies are necessary to determine whether these new mechanisms of action will impact our knowledge on endocrine control of inflammation and drug development.

## Author Contributions

All authors have made a substantial, direct, and intellectual contribution to the work and approved it for publication.

## Conflict of Interest Statement

The authors declare that the research was conducted in the absence of any commercial or financial relationships that could be construed as a potential conflict of interest.
